# Expression of Fascin and DNA Topoisomerase 2-Alpha in Breast Carcinoma: Correlation with Histological Subtypes and Other Prognostic Markers

**DOI:** 10.3390/ijms26073076

**Published:** 2025-03-27

**Authors:** Alberto Sánchez-Espinosa, José García-Rodríguez, Virginia Alonso-Aguirre, Jesús María Acosta-Ortega, Pablo Conesa-Zamora, José García-Solano, Ginés Luengo-Gil

**Affiliations:** 1 Group of Molecular Pathology and Pharmacogenetics, Pathology Department, Instituto Murciano de Investigación Biosanitaria (IMIB), Hospital Universitario Santa Lucía, 30202 Cartagena, Spain; mdsanchezespinosa@gmail.com (A.S.-E.); pepiscogr888@gmail.com (J.G.-R.); jesusm.acosta@carm.es (J.M.A.-O.); jgarcia652@ucam.edu (J.G.-S.); 2 Health Sciences Faculty, Universidad Católica de Murcia (UCAM), 30107 Guadalupe, Spain; 3 Department of Radiation Oncology, Hospital Universitario Santa Lucía, 30202 Cartagena, Spain; virginia.alonso2@gmail.com

**Keywords:** fascin, DNA topoisomerase 2-alpha, breast cancer, molecular subtypes, immunohistochemistry

## Abstract

Background/Objectives: Breast cancer is the most prevalent cancer in adult women. Currently, new therapies and protein determinations with prognostic value are under development. Fascin (encoded by the *FSCN1* gene) is an actin-binding protein that is critical for the development of cytoplasmic projections that are essential for tumor invasion. DNA topoisomerase 2-alpha (*TOP2A*) is a nuclear protein crucial for ATP-dependent breakage, passage, and rejoining of double-stranded DNA and cell division. Both proteins are associated with higher proliferation rates and worse prognosis in breast cancer and together can provide comprehensive information on prognosis and treatment response. Methods: We simultaneously assessed fascin expression and TOP2A/CEP17 DNA copy number ratios in various histological and molecular subtypes. Additionally, these markers were analyzed along with previously established diagnostic markers and other relevant clinical data. Results: Our series included 265 patients, four of whom were male, and all of which were diagnosed with breast carcinoma. Of the 265 patients initially included, sufficient material for analysis was available for 175 cases, as some samples were excluded because of insufficient tissue quantity, poor preservation, or lack of hybridization in certain assays. Immunohistochemical (IHC) expression of fascin, both in its aggregated form and by category, showed no association with the TOP2A gene alteration ratio. Fascin expression was significantly associated with histological subtype (*p* < 0.001), molecular subtype (*p* < 0.001), hormone receptor (HR) (*p* < 0.001), BCL2 (*p* = 0.003), Ki67 (*p* = 0.002), and histological grade (*p* < 0.001). TOP2A was significantly associated with molecular subtype (*p* = 0.041), Ki67 (*p* = 0.048), and histological grade (*p* = 0.033). In our study, molecular subtype (*p* = 0.037) emerged as an independent variable for the complete histological response to neoadjuvant treatment. Multivariate analysis linked pathological stage (*p* = 0.002) and estrogen receptor (ER) expression (*p* = 0.004) to overall survival (OS) and disease-free survival (DFS). Conclusions: No statistical relationship was evident between fascin expression (IHC) and the TOP2A copy ratio. The results of this study suggested that the mechanisms of increased cell proliferation associated with alterations in fascin and TOP2A are independent.

## 1. Introduction

Breast cancer is the most prevalent cancer among women globally [1], with a peak incidence at 40 years of age, varying geographically [2]. Risk factors include sex, age, ethnicity [3], family history [4], BRCA gene alterations [5], late menarche, early menopause [6], nulliparity, delayed first childbirth, shorter breastfeeding duration, contraceptive use, and hormone replacement therapies [7]. Regional screening is free for women aged 47–70 years, utilizing radiological techniques, such as contrast mammography, tomosynthesis, ultrasound, and nuclear magnetic resonance, with digital mammography as the standard. Mammary carcinoma, which originates in the ductal-lobular unit, is the most common malignant breast cancer. Samples were collected through core needle biopsy (CNB), vacuum-assisted biopsy (VAB), or incisional biopsies, and histological classification followed the “WHO Classification of Tumors” 5th edition, with most tumors classified as “no other subtype (NOS)” except for some such as mucinous carcinomas with differential histological findings.

Advancements and increased accessibility to molecular studies have refined the clinical classification of breast cancer by integrating molecular biology into oncological treatments [8]. The “Intclusters” classification [9] was developed by analyzing copy number and gene expression. The St. Gallen’s intrinsic classification in 2013 [10] was based on the IHC study (HR, Ki67, HER2), enabling its adoption in most centers. “Gene expression profile tests” [11] (e.g., MamaPrint™, OncotypeDx) are selectively used to assess metastasis recurrence risk with genetic scores of carcinogenesis-related genes, primarily those tied to proliferation and metastasis. In developed countries, breast cancer treatment is personalized and multidisciplinary [12], involving surgery, chemotherapy, immunotherapy, radiotherapy, and hormone therapy.

### 1.1. Fascin and Cancer

Fascin (*FSCN1*), a 55 kDa protein, cross-links at least 10 actin filaments to form bundles, thereby enhancing cell rigidity and overcoming membrane resistance [13]. It stabilizes cytoplasmic protrusions such as filopodia [14]. Located on chromosome 7p22.1, fascin is regulated by serine 39 phosphorylation via protein kinase C [15], and is most abundant in the CNS and dendritic cells [16]. Typically absent in most normal epithelial tissues, it is found in dendritic, endothelial, and mesenchymal cells, neuronal tissues, and various tumors, including breast cancer [17]. Overexpression is correlated with aggressive tumors and poor outcomes, aiding in cancer cell migration and invasion [18]. No approved anti-fascin drugs exist, but several compounds are under trial [19], including direct inhibitors (macroketones [20], thiazole derivatives [21], imipramine [22,23,24,25], and raltegravir [26]) and indirect inhibitors (melatonin [27], chalcone [28], curcumin [29], and Elaeagnus angustifolia extracts [30]).

### 1.2. Topoisomerase II Alpha

Topoisomerase II alpha (*TOP2A*, 17q21.2), a 170 kDa nuclear homodimer, belongs to the DNA topoisomerase family. It acts on double-stranded DNA, requiring ATP and Mg2+ without chemically modifying the DNA. *TOP2A* expression peaks in the G2/M phase of the cell cycle, creating temporary breaks in one DNA strand (G segment) to allow another strand (T-transported segment) to pass through before resealing the break [31], facilitating decatenation and the unwinding of genetic material essential for replication, transcription, and mitosis. Catalytic inhibitors [32] inhibit the enzyme’s main activity by blocking ATP hydrolysis after strand passage without increasing TOP2A-DNA covalent complexes (TDCC), whereas TOP2A poisons [32] increase TDCC, inducing apoptosis by causing genomic breaks. Anthracyclines intercalate into DNA, blocking enzymatic function, despite cardiotoxicity. *TOP2A* gene amplification in cancer cells leads to overexpression, increased proliferation, and aggressiveness, and affects chemotherapy sensitivity and prognosis [33].

This study investigated fascin and *TOP2A* expression in breast cancer and their relationship with clinical outcomes. Both proteins are associated with tumor aggressiveness and poor prognosis but have not been studied in detail. Using IHC for fascin and fluorescence in situ hybridization (FISH) for *TOP2A* gene copy number in the same samples, we sought correlations with the established prognostic factors. We also assessed whether the combined analysis offered more comprehensive prognostic information than individual markers. Understanding their interactions could provide insights into breast cancer biology and identify potential therapeutic targets.

## 2. Results

### 2.1. Expression of Fascin and TOP2A by Categories and Grouping

IHC fascin expression for both intensity and percentage of stained cells was predominantly in category A at 54% and 61.1%, respectively. The lowest intensity value percentage was observed in category D (5.6%) and for intensity in category C (9.1%). Dichotomous expression showed a predominance of “Low-grade” (77.4%) over “High-grade” (22.6%). A significant correlation was found between higher expression and fascin intensity (*p* < 0.001).

In 265 patients, adequate *TOP2A* evaluation was not possible in 90 cases because of insufficient cells, lack of hybridization, or unavailability of the material. FISH analysis of *TOP2A* was performed in 175 samples, with a mean *TOP2A* ratio of 1.28. The results indicated that the normal category (*n* = 136) was more common than the altered category (*n* = 39), with gain (*n* = 21) appearing more frequently than the sum of the deletion and amplification cases (*n* = 6 and *n* = 12, respectively).

Statistical analysis of the relationship between grouped fascin expression and individual/grouped *TOP2A* ratio categories in 175 patients showed no significant difference (*p* = 0.893). Similarly, ANOVA revealed no relationship between fascin expression/intensity and the *TOP2A* ratio (*p* = 0.650 and *p* = 0.167, respectively).

### 2.2. Fascin Expression in Different Histological and Molecular Subtypes of Breast Cancer

Our series revealed a statistically significant correlation between histological subtype and fascin expression (*p* < 0.001). The “high-grade” fascin expression in subtypes was as follows: NOS carcinoma (formerly ductal) 41/133 (30.8%), invasive lobular carcinoma 5/47 (10.6%), mucinous carcinoma 1/24 (4.2%), tubular carcinoma 1/20 (5%), infiltrating papillary carcinoma 0/15 (0%), carcinoma with medullary pattern 6/12 (50%), micropapillary carcinoma 1/6 (16.7%), apocrine carcinoma 2/5 (40%), and adenoid cystic carcinoma 3/3 (100%). The molecular subtype was also significantly associated with fascin expression (*p* < 0.001). The “high-grade” expression distribution among molecular subtypes was as follows: luminal A 8/84 (9.5%), luminal B HER2− 16/109 (14.7%), luminal B HER2+ 5/19 (26.3%), HER2+ 0/2 (0%), and triple-negative breast cancer (TNBC) 27/39 (69.2%). Our series indicated “high-grade” expression in TNBC and luminal B HER2−, while HER2+ was less significant due to limited representation. Given the low number of HER2+ cases, the statistical power to detect meaningful associations in this subgroup was limited, and the results should be interpreted with caution. Future studies with larger HER2+ cohorts are warranted to validate these findings.

### 2.3. TOP2A/CEP17 Ratio in Different Histological and Molecular Subtypes of Breast Cancer

The *TOP2A*/CEP17 ratio categories exhibited no significant differences (*p* = 0.610) in the histological subtypes (Table 1). No amplification or deletion was observed in adenoid cystic carcinoma, apocrine carcinoma, micropapillary carcinoma, infiltrating papillary carcinoma, or tubular carcinoma. The NOS and medullary pattern types included all the recorded categories. Subtypes with *n* < 5 were consolidated into “Normal” and “Altered” categories, yielding similar results (*p* = 0.188). A significant relationship between *TOP2A* and molecular subtype was found with dichotomous *TOP2A* grouping (*p* = 0.041), with “Altered” expression in luminal A 6/48 (12.5%), luminal B HER2− 20/75 (26.7%), luminal B HER2+ 8/17 (47%), HER2+ 0/1 (0%), and TNBC 4/24 (16.7%).

### 2.4. Fascin Expression and TOP2A Ratio Concerning Histological Grade and Pathological-Tumor Stage

Both Scarff–Bloom–Richardson grade (SBR) evaluations by grouped category showed significant relationships (*p* < 0.001 and *p* = 0.003, respectively). SBR Grade 1 had “High-grade” fascin expression in 7/80 cases and “Altered” *TOP2A* in 9/52. For SBR Grade 2, the values were 29/130 and 56/84, respectively, and for SBR Grade 3, they were 22/49 and 2/34, respectively. SBR Grade 2 exhibited the highest *TOP2A* alteration, whereas Grade 3 had the highest fascin expression.

Pathological stage (pTNM) was assessed in patients with breast cancer without neoadjuvant treatment. Fascin was evaluated in pT (*n* = 158), pN (*n* = 175), and pM (*n* = 173), while *TOP2A* was assessed in pT (*n* = 123), pN (*n* = 116), and pM (*n* = 115). No statistically significant relationships were found between these variables (*p* > 0.05). Clinical stage (cTNM) evaluations also showed no statistical significance for fascin expression or *TOP2A* ratio (*p* > 0.05). Similarly, grouped tumor stages (I-IV) showed no significant differences (*p* > 0.05).

### 2.5. Expression of Fascin and TOP2A Ratio Concerning Other Clinicopathological Variables

The mean age of the patients was 64.69 years (range 25–99 years), with 261 women and 4 men. Of them, 182 were postmenopausal at the time of the study. Neither fascin expression nor *TOP2A* ratio was associated with age (*p* = 0.124; *p* = 0.335), sex (*p* = 0.910; *p* = 0.643), or menopause (*p* = 0.144; *p* = 0.923).

Regarding the type of surgery, conservative/local mastectomy was predominant in 49.8% (*n* = 118) of the patients who underwent radical mastectomy, which represented 19.4% (*n* = 46).

**Figure 1:**
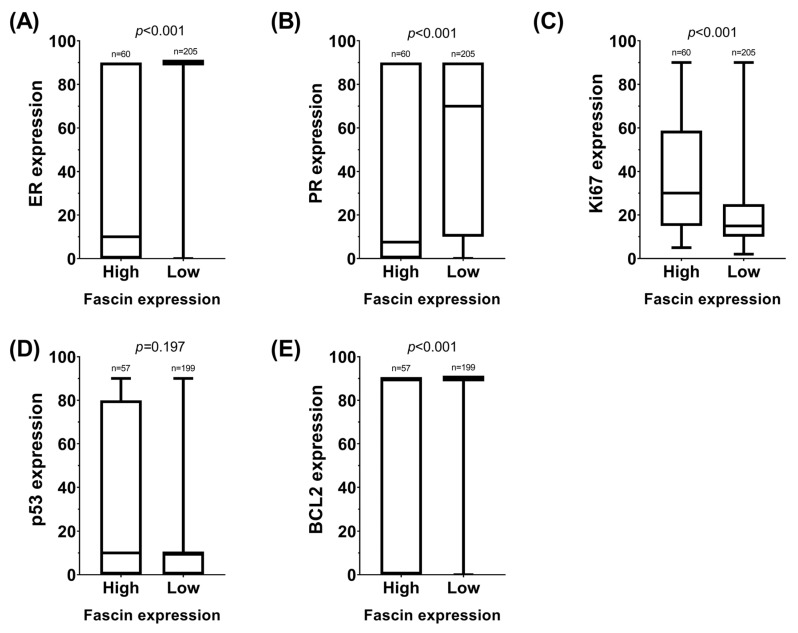
Box plots illustrating the distribution of routine immunohistochemical (IHC) variables used in breast cancer diagnosis, stratified by fascin expression levels. Panel (**A**) represents estrogen receptor (ER) expression; panel (**B**), progesterone receptor (PR) expression; panel (**C**), Ki67 expression; panel (**D**), p53 expression; panel (**E**), BCL2 expression.

The relationship between fascin and TOP2A and the IHC expression of E-cadherin, CK19, and HER2+ was considered. None of the groups showed statistical significance (*p* > 0.05). Similarly, Student’s *t*-tests were performed to analyze the probable relationship between the expression of usual IHC markers in breast cancer diagnosis (ER, progesterone receptor PR, Ki67, p53, and BCL2) and dichotomously grouped IHC expression of fascin to obtain a box plot (Figure 1). For a more accurate evaluation, the ER, PR, p53, and BCL2 levels were also dichotomized, considering two possibilities: 0% expression (negative) or ≥1% (positive). Ki-67 was associated with fascin and TOP2A (*p* = 0.002 and *p* = 0.048, respectively). All the data are presented in Table 2.

### 2.6. Correlation Analysis Between Continuous Variables and ROC Curves

The continuous variables analyzed were ER, PR, Ki67, p53, BCL2, and the *TOP2A* ratio. Pearson’s correlation yielded a *p*-value of < 0.001 for all variables except *TOP2A*. ER, PR, and BCL2 expressions were positively correlated, whereas they were inversely correlated with p53 and Ki67 expressions. The p53 and Ki67 levels were positively correlated. ROC curves for these variables indicated a significant result only for Ki67, with an AUC of 0.822.

### 2.7. Study of pCR in Relation to Fascin/TOP2A and Other IHC Markers

Assessment of pathological complete response (pCR) with fascin expression showed an OR of 2.7 (95% CI = 0.664–10.704; *p* = 0.167), indicating no statistically significant relationship. Insufficient FISH detection of TOP2A in patients with pCR prevented a similar analysis of TOP2A. Univariate binary logistic regression (Table 3) revealed significant differences in ER (*p* = 0.005), BCL2 (*p* = 0.010), Ki67 (*p* = 0.003), histological grade SBR (*p* = 0.004), clinical stage (*p* = 0.046), molecular subtype (*p* = 0.009), and TNBC (*p* = 0.021). OR > 1 for ER, clinical stage, BCL2, and TNBC; OR < 1 for histological grade, SBR, Ki67, and molecular subtype. A *p* > 0.05 was found for PR, p53, E-cadherin, menopause, age, and histological subtype. In the multivariate analysis, ER, Ki67, and BCL2 were excluded due to significant positive correlations (Pearson’s correlation coefficient). Only molecular subtype remained an independent prognostic variable for pCR (*p* = 0.037).

### 2.8. Overall Survival Analysis Concerning Fascin/TOP2A and Other IHC Markers

The evaluation of vital status at the end of the study (*n* = 257) showed a 10.5% mortality rate, with 8.6% not disease-free at death. Tumor recurrence analysis revealed no significant differences in fascin (*n* = 17) or TOP2A ratios (*n* = 13) (*p* = 0.514 and *p* = 0.568, respectively). By the end of the follow-up period, 78.2% of the patients were disease-free. OS was not significantly associated with fascin expression or *TOP2A*/CEP17 ratio (*p* = 0.853 and *p* = 0.181, respectively). The Mantel–Cox test indicated significance in histological subtype (*p* = 0.006), tumor stage (*p* = 0.002), ER (*p* = 0.020), BCL2 (*p* = 0.041), and age over 65 years (*p* ≤ 0.001). Cox regression analysis (Table 4) identified the pathological stage (*p* = 0.002) as the sole independent variable in the OS multivariate analysis.

### 2.9. Disease-Free Survival Analysis Concerning Fascin/TOP2A and Other IHC Markers

Fascin expression (*p* = 0.188) and the *TOP2A* ratio (*p* = 0.954) were not associated with DFS. Univariate analysis of other IHC markers and clinical data showed statistical significance only for molecular subtype (*p* < 0.001) and ER (*p* = 0.01). In multivariate Cox regression analysis, only ER (*p* = 0.004) emerged as an independent DFS variable.

### 2.10. Other Unassessed Histological Markers

Tumor necrosis with fascin expression or the *TOP2A* ratio was not assessed, nor were the changes between initial CNB and post-neoadjuvant or metastatic disease.

## 3. Discussion

Our patients had a mean age of 64.66 years, notably higher than that in the Iran, Korea, and Turkey series, with mean ages of 48.4, 48.2, and 53.73 years, respectively [34,35]. We found fewer HER2+ cases (0.8%) than 31.1% in previous studies [35]. The distribution of luminal B cases was comparable when luminal B HER2+ and luminal B HER2− were combined. Our study included both sexes and all ethnicities, unlike some studies that focused on specific ethnic groups, such as African descent, which showed a higher incidence of TNBC [36]. The small number of men (*n* = 4) reflects the 1% prevalence of male breast cancer; however, fascin and *TOP2A* expression variability in men remains uncertain. Surgery type, adjuvant treatment, and recurrence were not considered as variables in any study. The percentage of radical mastectomies (19.4%) was consistent with that reported in other studies [34]. Our study found no significant associations between fascin expression or *TOP2A* alterations and OS, DFS or tumor recurrence. These findings contrast with previous reports suggesting a prognostic role for these markers in breast cancer progression and treatment response. Several factors may explain this discrepancy. First, sample size limitations may have reduced statistical power, particularly in underrepresented subgroups like HER2+, where a post hoc power analysis confirmed that our study had insufficient power (<80%) to detect moderate effect sizes. Second, methodological differences across studies, including variability in fascin quantification methods, antibody selection, and positivity thresholds, could have influenced the results. Third, the biological complexity of fascin and *TOP2A* suggests that their role in breast cancer may depend on indirect mechanisms or interactions with other molecular pathways not fully captured by IHC or FISH.

Despite these limitations, our study confirmed that molecular subtype and pathological tumor stage were significantly associated with DFS and OS, reinforcing their established clinical relevance. Given the lack of prognostic significance of fascin and *TOP2A* in our cohort, these markers may have limited immediate application for risk stratification or treatment selection in routine clinical practice. However, their known involvement in tumor invasion and chemotherapy response warrants further investigation, particularly in specific molecular subtypes. Future studies should focus on integrating fascin and *TOP2A* expression into multigene classifiers or combining them with functional assays to refine their clinical utility.

Additionally, a key limitation of our study is the lack of internal validation, such as bootstrapping or cross-validation, which could have strengthened the robustness of our statistical models. Without these techniques, there is a risk of overfitting, particularly in subgroups with small sample sizes, such as HER2+. Although our models were adjusted for potential confounders, external validation in an independent cohort is necessary before these findings can be considered clinically applicable. The retrospective nature of this study and the underrepresentation of specific molecular subtypes further highlight the need for prospective, multicenter validation studies to confirm these observations and refine the prognostic and predictive value of fascin and *TOP2A* alterations in breast cancer.

In our study, 22.6% of samples showed “High-grade” fascin expression, comparable to other studies [35], but contrasting with a Japanese study [37] in luminal A and B HER2+ subtypes, aligning with other research [34]. Discrepancies may be due to population-specific variants or differences in the quantification systems. Variability in fascin expression quantification methods complicates comparisons, with studies lacking clarity on thresholds and current consensus on a method of evaluation, considering cytoplasmic positivity [35], or using arbitrary cut-off points without intensity levels. In our method, the percentage of expression is assessed by calculating the total tumor area in the sample while excluding stromal tissue and non-evaluable regions, such as artifacts and necrotic areas. Staining intensity is classified as follows: absent (no detectable staining); strong (intensity comparable to that of blood vessels); moderate (slightly less intense yet similar to blood vessel staining); or weak (detectable but not meeting the criteria for the other categories). We consider this a straightforward quantification method for expert pathologists, similar to the assessment of HER2 in breast or gastric cancer. Various studies [37,38,39] employ different scoring systems, but we argue that even moderate, extensive fascin expression should be classified as “High-grade”. Our method, dividing expression into “High-grade” and “Low-grade” subgroups, facilitates broader comparisons. Distinguishing true staining from background noise is challenging, and we support the use of internal vascular controls, eliminating the need for external controls. mRNA studies may include non-tumor cells that exhibit high fascin expression at the tumor front [40]. The monoclonal mouse clone (clone 55k-2; Dako, Santa Clara, CA, USA) was selected based on previous studies. Our results align with most studies, showing an inverse correlation between fascin and RH expression [22,34,35,38,40,41,42,43], with Ki67 significance in some studies [37,40,42]. p53 showed no correlation with fascin expression, consistent with other studies [40], unlike RH, BCL2, and Ki67, which correlated using Pearson’s test.

Our results demonstrated a correlation between fascin and both histological and molecular subtypes, which was not observed in all series [35]. “High-grade” fascin expression was prominent in rare histological subtypes, such as the medullary and apocrine subtypes (6/6 and 2/3 cases, respectively), suggesting that these subtypes should be included in future research. Fascin expression is higher in TNBC (69%) than in Luminal A (3.2%) and is associated with poor prognosis [34,37,39,42,44]. In African American women, fascin’s sensitivity and specificity for predicting TNBC were 82.1% and 80%, respectively [36]. Fascin expression was not significantly related to HER2 alterations (*p* = 0.050) [41] but was associated with advanced disease histology, including tumor size [35], lymph node invasion [43], lymphatic invasion [45], and extensive in situ components [44]. Higher fascin expression correlates with higher SBR grades [34,40,41,43], suggesting a link between fascin and poor prognosis and tumor progression. The average TOP2A/CEP17 ratio was 1.28, and dichotomous classification was used for comparison. An Egyptian study found a significant relationship between CEP17 polysomy and TOP2A copy number alterations [46].

*TOP2A* expression can be assessed using different methods: IHC [47], mRNA expression via microarray, reverse transcription polymerase chain reaction (RT-PCR), chromogenic in situ hybridization (CISH), and FISH *TOP2A*/CEP17 [48,49,50,51]. TOP2A amplification and overexpression should not be considered synonymous because cellular signals can increase protein expression independently of copy number and association with other cell proliferation markers [52]. Similarly, although not observed in our study, a CEP17 ratio ≥ 3 should be considered indicative of polysomy. In these cases, since the observed overexpression may not reflect genuine *TOP2A* gene amplification, such cases should either be excluded or classified as a separate category. To maintain cohort representativeness despite the loss of 90 cases, we performed comparative analyses that revealed no significant differences in age, gender, molecular subtype, or histological subtype between the included and excluded patients (all *p* > 0.05). We recommend prospective studies that allow for improved DNA preservation for FISH assays. The amplification and deletion rates were approximately 7% and 3.4%, respectively. A Canadian series [53] with 438 patients reported slightly higher rates (12% and 6%, respectively). Similar rates were found in Spain, the U.S., and Taiwan, with 8.6% amplification in 232 cases [54], 9.4% in 153 patients [55], and 9.8% in 296 cases [56]. Deletion rates were 2.7% in a Taiwanese study, 5.4% in an American study, and 2.1% in a Korean study [57]. Comparing these results with those of other studies using IHC for *TOP2A* evaluation showed higher positivity rates, with 29.3% and 55.8% in a Japanese study [51]. The *TOP2A* and HER2 (*ERBB2*) genes are located close to chromosome 17q12-21. A FISH study [56] found an 8.4% co-amplification tendency, while other studies [58] reported 3.7%. The literature has described *TOP2A* deletion with HER2 amplification [56]; however, our results did not show co-amplification or *TOP2A* deletion with HER2 amplification. The prevailing scientific view suggests that the amplification of one does not imply amplification or modification of the other, despite their chromosomal proximity [59].

Our findings linked increased TOP2A gene copies to a higher Ki67 percentage, similar to another study [54] and others evaluating TOP2A using IHC [49,51,60,61] and CISH [33,62]. *TOP2A* ratio was related to the histological grade measured by SBR, primarily in Grade 2 cases, in which 50% (28/56) showed alterations. SBR’s statistical association of SBR with TOP2A has been noted in other IHC [49,60,63] and FISH [56] studies. Ki67’s role as a cell proliferation marker and the necessity of *TOP2A* protein for this process suggest a correlation in rapidly growing tumors. Notably, Ki67 and SBR expression were significantly associated with fascin expression. Studies have shown a statistical relationship between high expression and IHC/*TOP2A* copy number alteration [47,48,51,53,54,56], reduced HR expression [54,62,64], tumor stage [48], size [53,61,64], grade [47], and positive lymph nodes [47]. Contradictory findings exist for the latter, with some studies linking lower *TOP2A* levels [53]. Our analysis demonstrated a significant relationship with molecular subtype, with a 36.4% *TOP2A* alteration in the luminal B HER2− subtype, similar to IHC studies [51], but differing drastically when evaluated by FISH (0.3%). The limited HER2+ subtype representation hindered exhaustive evaluation, although a Spanish study [54] reported 66% amplification in the HER2+ subgroup via IHC and FISH.

Few studies have conducted inferential analyses based on histological subtypes of breast cancer. One study found no relationship, including only the NOS, lobular, and mixed subtypes [54]. A Croatian study [60], grouping subtypes into “NOS” and “OTHERS”, obtained significant results via IHC evaluation of TOP2A with a 37% cutoff. A Portuguese study [33], without statistical significance, also grouped histological subtypes dichotomously. German [58] and Turkish [64] studies performed histological subdivisions but no specific inferential analysis. Notably, our series, despite partial representation of various histological subtypes, showed no significant results, although *TOP2A* was altered in 91% of mucinous carcinomas and 33% of lobular carcinomas. A Japanese study [61] evaluated TOP2A levels before and after treatment in partial responses and found increased expression after treatment. A Turkish study [64] found a significant positive association between geographic necrosis, nipple involvement, and TOP2A levels using IHC. Our exploratory analysis found no correlation between *TOP2A*, CK19, and E-cadherin, similar to the fascin analysis.

The relationship between *TOP2A* evaluated by FISH and age [51,53] (except for a Taiwanese study with a 50-year cut-off [56]), menopause [49], and sex showed no statistical significance. p53 expression was not significant in our series, but was observed in other studies [54,65]. Comparing histological grades among different studies is complicated as some studies only considered the nuclear grade. Our study observed a positive correlation between BCL2 and ER expression, similar to that observed in a Japanese study [66]. BCL2 expression is an independent prognostic marker indicating poor prognosis when associated with negative HR, but tumors positive for BCL2 tend to have a better prognosis because of the generally better prognosis of ER-positive tumors. Studying fascin expression along with other proteins is a contemporary approach in breast cancer research. GATA3, a nuclear protein, influences genomic transcription and is expressed in breast and urothelial cancer. BRMS1 acts as a tumor suppressor. GATA3 is associated with good prognostic factors, but fascin+/GATA3− expression indicates worse overall survival and lower SLE [67]. Tumors with fascin+/BRMS1− expression had a higher histological grade, HR negativity, and lower lymph node metastasis [43]. A Croatian study [68] found an association between TOP2A expression and testicular tumor antigens MAGE-A10 and NY-ESO in TNBC. Simultaneous evaluation [65] of MAP-tau and TOP2A in patients with positive lymph nodes did not predict a better response to anthracyclines or taxanes. There is a relationship between *TOP2A* levels and PTEN gene deletion in TNBC cases [61]. A Turkish study [64] showed a significant association between TOP2A and RacGAP1, which is a protein involved in cell proliferation and differentiation.

## 4. Materials and Methods

This retrospective, anonymized, descriptive observational study was conducted at the Cartagena University Hospital Complex (Spain) from 2013 to 2023 on patients with a pathological breast carcinoma diagnosis via CNB.

### 4.1. Selection Criteria

The primary inclusion criterion was a confirmed breast carcinoma diagnosis at our center with sufficient sample quantity for additional fascin and *TOP2A* determinations. Exclusion criteria included consultation cases, cases without residual lesions in the paraffin block, and cases lacking sufficient clinical data or prior immunohistochemical or molecular diagnostics. Age, sex, and race were not restricted in this study. A total of 265 patients were initially included, of which 175 had sufficient material for analysis, while others were excluded due to insufficient tissue, poor preservation, or unsuccessful hybridization.

The cohort was nonconsecutive, and the selection was based on material availability, which may have introduced sample selection bias. Additionally, variability could arise from differences in fixation times, preanalytical conditions affecting antigen preservation, and potential disparities in tissue-processing techniques between samples. These factors may contribute to minor inconsistencies in staining intensity and expression levels. However, we implemented internal quality controls to minimize these effects.

### 4.2. Histological and Molecular Subtype Classification, Histological Grade, and Slide Preparation for Additional Studies

Histological subtypes were classified per the 5th edition of the WHO for Breast Cancer. Molecular subclassification was based on IHC studies [12]. The Nottingham combined histological grade [69], modified by the Elston–Ellis system from the Scarff–Bloom–Richardson grading system, quantifies gland formation, nuclear atypia, and mitotic count. Three new sections were prepared for each selected case: Hematoxylin and Eosin (H&E) to assess residual tumor, IHC for fascin, and FISH for *TOP2A*. The samples were fully utilized without discarding or trimming the CNB. To optimize resources, four determinations per slide were created. For the FISH study of *TOP2A*, paraffin sections were prepared 24–48 h before hybridization, placing samples in the lower two-thirds of the slide to improve the hybridization rates.

### 4.3. Fascin and DNA Topoisomerase 2-Alpha Quantification

**Figure 2:**
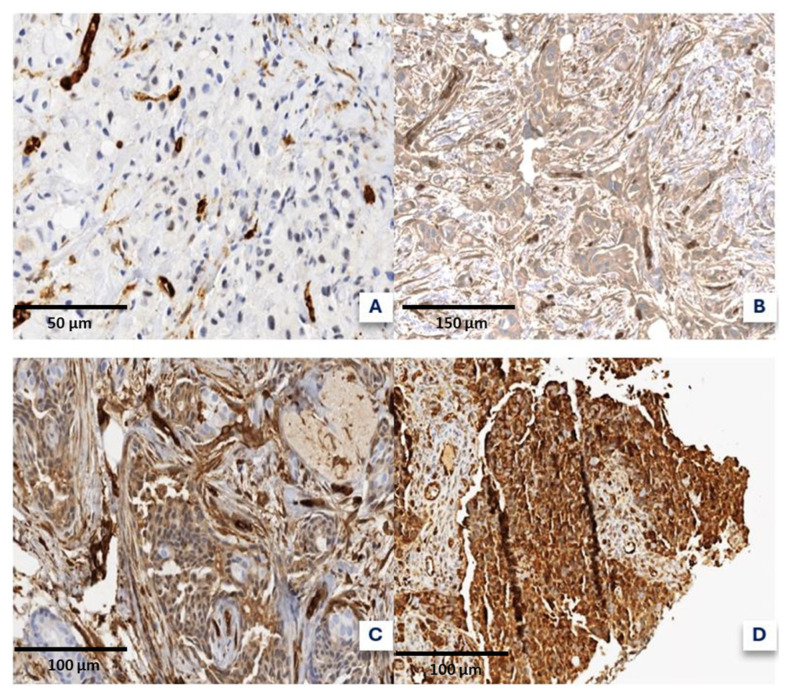
Different levels of fascin expression were observed in the four categories. (**A**) No staining. (**B**) Weak. (**C**) Moderate. (**D**) Strong.

Fascin expression was assessed by IHC using the rabbit monoclonal antibody 55k-2 (Cell Marque™, Rocklin, CA, USA) processed in the BenchMark Ultra system (Roche Ventana^®^, Basel, Switzerland). Quantification followed a semi-quantitative method adapted from a Turkish study [44], considering both intensity and extent of expression. The expression percentages were categorized as follows: A (<10%), B (11–50%), C (51–75%), and D (76–100%). Signal intensity was classified as A (no staining), B (weak), C (moderate), and D (strong) (Figure 2). Fascin expression was then reclassified as high- or low-grade based on a threshold adapted from prior research and refined for our dataset. Specifically, samples with at least a C (51–75%) or D (76–100%) in either the intensity or percentage categories were classified as “high-grade”. This approach aligns with previously published methodologies that define high fascin expression as moderate-to-strong staining in a significant proportion of tumor cells. To ensure repeatability and consistency, two independent pathologists with expertise in breast cancer independently evaluated the slides. Any discrepancies in scoring were resolved by a third reviewer through a consensus meeting. Additionally, internal vascular controls were used in each case to distinguish true-positive staining from background noise, following previously validated methodologies.

For *TOP2A*, the CT-PAC008-10-OG probe (CytoTest^®^, Rockville, MD, USA) was used in the MD-Stainer hybridizer (Vitro Master Diagnostica^®^, Granada, Spain) on 4-micron sections. A semi-automated protocol included pretreatment, enzymatic digestion, manual probe dispensing, DNA denaturation, and 16 h hybridization, with post-hybridization washes using Tris-Buffered saline. Quantification was performed by a pathologist experienced in FISH studies using a fluorescence microscope (Olympus BX53, Tokyo, Japan). The TOP2A/CEP17 copy number ratio was recorded in at least 50 tumor cells and subclassified based on cutoff points reported in the literature [70]: deletion (ratio < 0.7), normal (ratio 0.7–1.3), gain (ratio > 1.3 and <2), and amplification (ratio ≥ 2). For the analysis of the TOP2A gene copy number, the CEP17 probe (Chromosome Enumeration Probe 17) was used as a reference to normalize the TOP2A signal. CEP17 ensures accurate ratio calculations by accounting for variations in the chromosome 17 copy number, which may result from polysomy or other chromosomal abnormalities. This normalization is essential for distinguishing true TOP2A amplification or deletions from background chromosomal variations, thereby enhancing the reliability of the results. This allows for the assessment of molecular status and distinguishes between overexpression or negativity in immunohistochemical studies without genomic alterations.

### 4.4. Other IHC Determinations

E-Cadherin (EP700Y, Cell Marque™, Rocklin, CA, USA, USA) and CK19 (A53-B/A2, Cell Marque™, Rocklin, CA, USA, USA) were analyzed dichotomously (yes/no). The other markers were quantified as a percentage: estrogen receptor ER (SP1, Roche Ventana^®^, Basel, Switzerland), progesterone receptor PR (1E2, Roche Ventana^®^, Basel, Switzerland), Ki67 (30-9, Roche Ventana^®^, Basel, Switzerland), p53 (DO-7, Ventana^®^, Basel, Switzerland), and BCL2 (SP66, Ventana^®^, Basel, Switzerland). HER2 status was assessed by IHC (4B5, Roche Ventana^®^, Basel, Switzerland). A 2+ result necessitated further classification using SISH (HER2 Dual ISH, Roche Ventana^®^, Basel, Switzerland) or FISH (MAD-001FA, Vitro Master Diagnostica^®^, Granada, Spain). The IHC and SISH sections were 3–5 µm and 4 µm thick, respectively, for FISH. All procedures except FISH were performed using BenchMark Ultra (Roche Ventana^®^, Basel, Switzerland).

### 4.5. Clinical and Prognostic Variables

The clinical history variables included sex, age, menopause status, neoadjuvant and adjuvant treatments (type specified), pathological response to neoadjuvant treatment, surgical details (type and date), biopsy-to-surgery interval, tumor recurrence (date and type), clinical and pathological TNM staging according to the American Joint Committee on Cancer (AJCC) 8th edition [71], vital status at study conclusion, and date of death.

### 4.6. Statistical Analysis

SPSS Statistics 21 software (IBM, Armonk, New York, USA) was used for the descriptive and inferential analyses. Quantitative variables included absolute and relative frequencies, while qualitative variables included the mean, median, range, and standard deviation. Statistical significance for all studies was set at *p* < 0.05, using a two-tailed contrast. Pearson’s chi-squared test, continuity correction for 2 × 2 tables, and likelihood ratios were used to determine the correlation between nominal variables, with proportions compared using the chi-squared test. Spearman’s correlation coefficient analyzed correlations between continuous variables.

Overall and disease-free survival were assessed using the Kaplan–Meier test, with group comparisons using the Mantel–Cox test. Cox regression analysis was used for multivariate survival analysis. Binary logistic regression was employed to assess associations between predictor variables and pCR. The discriminative capacity of dichotomous diagnostic tests for pCR was evaluated using ROC curves, providing an estimation of the predictive value of the models.

The alpha and beta errors were controlled by setting a significance level of *p* < 0.05 to mitigate type I (alpha) error, minimizing the likelihood of incorrectly rejecting null hypotheses. To address type II (beta) errors, power analyses were conducted during the study planning phase to estimate the sample size necessary to detect meaningful differences or associations. Additionally, a post hoc power analysis was performed to assess the statistical robustness of findings in subgroups with limited representation, particularly HER2+ cases. This analysis revealed that the statistical power for detecting moderate effect sizes in the HER2+ subgroup was below 80%, suggesting that the results for this molecular subtype should be interpreted with caution.

Multivariate models were constructed by including variables that demonstrated statistical significance in univariate analyses (*p* < 0.05). To account for potential confounders, we initially evaluated the impact of key clinical and pathological variables, including age, menopausal status, tumor size, histological grade, molecular subtype, and treatment type (neoadjuvant vs. adjuvant therapy). Stepwise regression was used to retain only those variables that contributed significantly to model performance, with pathological tumor stage and ER status emerging as independent predictors of survival outcomes. Other variables, such as age and BCL2 expression, were excluded due to a lack of statistical significance in multivariate models. Furthermore, we performed correlation analyses to identify collinearity between variables, which led to the exclusion of highly correlated factors (e.g., Ki67, BCL2, and ER) to avoid redundancy.

## 5. Conclusions

This study is the first to investigate the combined expression of fascin and *TOP2A*/CEP17 ratio in breast cancer. No relationship was observed between fascin expression and alterations in the *TOP2A*/CEP17 ratio. Fascin is associated with HR absence or reduction, elevated Ki67, low BCL2 levels, higher histological grade, and poor prognosis of molecular subtypes, such as TNBC. The lack of standardized fascin quantification complicates comparisons between studies. *TOP2A* is related to the molecular subtype, histological grade, and high Ki67 expression. Multivariate analyses linked pathological stage with overall survival and ER with DFS. The molecular subtype is an independent marker of the response to neoadjuvant treatment.

### Study Limitations

The retrospective nature of this study introduces inherent biases, including patient selection bias, reflected in the heterogeneity of the cohort. A key limitation is the exclusion of 90 out of 265 cases (34%) from the TOP2A analysis due to insufficient material. Although a comparison of baseline characteristics between the included and excluded patients showed no significant differences in age, sex, menopausal status, histological subtype, or fascin expression, cases with a higher histological grade and more advanced pathological stage were slightly overrepresented (*p* < 0.05). This may reduce the generalizability of our findings to lower-stage breast cancer cases, warranting caution when extrapolating our conclusions. Furthermore, certain molecular subtypes, such as HER2+, were underrepresented compared to the expected prevalence of 10–20%, potentially limiting the statistical power to detect meaningful associations in these subgroups. Indeed, a post hoc power analysis confirmed that our study had insufficient power (<80%) to detect moderate effect sizes in HER2+ tumors, which may explain the lack of significant prognostic associations. Another limitation is that our statistical models did not include treatment-related variables (e.g., neoadjuvant chemotherapy, hormone therapy, or surgical approach) as potential confounders. While key clinical and pathological variables were considered, treatments may influence tumor biomarker expression and should be integrated into future analyses. Additionally, the lack of internal validation, such as bootstrapping or cross-validation, may impact the robustness and reproducibility of our statistical models, increasing the risk of overfitting, particularly in smaller subgroups. While our models were adjusted for potential confounders, external validation in an independent cohort is necessary to confirm their clinical relevance. Future prospective, multicenter studies with well-balanced cohorts would help assess the prognostic and predictive value of fascin and *TOP2A* alterations in breast cancer. Additionally, the lack of biological experiments prevents further mechanistic insight into the observed associations, limiting our ability to fully elucidate their role in tumor progression and treatment response. Addressing these limitations will be essential to strengthening the clinical applicability of our findings and guiding future research in this field.

## Figures and Tables

**Table 1:** TOP2A expression.

Pearson’s χ² *p* = 0.610		TOP2A Expression				Total
		Amplif	Gain	Normal	Deletion	
**Histological subtypes**	Adenoid cystic	0	0	2	0	2
	Apocrine	0	0	5	0	5
	Infiltrating lobular	3	2	15	0	20
	Medullary	0	0	8	1	9
	Micropapillary	0	1	3	0	4
	Mucinous	3	6	11	1	21
	NOS (Not Otherwise Specified)	6	8	72	4	90
	Infiltrating papillary	0	2	10	0	12
	Tubular	0	2	10	0	12
Total		12	21	136	6	175

**Table 2:** Clinical and immunohistochemical (IHC) variables in relation to fascin expression and *TOP2A*/CEP17 ratio. Variables with statistically significant differences are highlighted: Ki67 (*p* = 0.002 for fascin expression, *p* = 0.048 for TOP2A ratio), histological subtype (*p* < 0.001 for fascin expression), molecular subtype (*p* < 0.001 for fascin expression, *p* = 0.041 for *TOP2A* ratio), and BCL2 (*p* = 0.003 for fascin expression). The significance levels indicate the association of these variables with fascin expression or *TOP2A* alterations.

	Fascin			TOP2A		
Characteristic	High *n* (%)	Low *n* (%)	*p*	Normal *n* (%)	Altered *n* (%)	*p*
Molecular subtype			<0.001			0.041
Luminal A	8 (14)	78 (39)		42 (33)	6 (16)	
Luminal B HER2−	16 (29)	93 (47)		55 (43)	20 (53)	
Luminal B HER2+	5 (9)	14 (7)		9 (7)	8 (21)	
HER2+	0 (0)	2 (1)		1 (1)	0 (0)	
TNBC	27 (48)	12 (6)		20 (16)	4 (10)	
Histological subtype			<0.001			0.188
Adenoid cystic	3 (5)	0 (0)		2 (2)	0 (0)	
Apocrine	2 (3)	3 (2)		5 (4)	0 (0)	
Invasive lobular	5 (8)	42 (21)		15 (11)	5 (13)	
Medullary	6 (10)	6 (3)		8 (6)	1 (3)	
Micropapillary	1 (2)	5 (2)		3 (2)	1 (3)	
Mucinous	1 (2)	23 (11)		11 (8)	10 (25)	
NOS (Not Otherwise Specified)	41 (68)	92 (45)		72 (53)	18 (46)	
Invasive papillary	0 (0)	15 (7)		10 (7)	2 (5)	
Tubular	1 (2)	19 (9)		10 (7)	2 (5)	
ER			<0.001			0.166
Positive	31 (52)	185 (90)		109 (80)	35 (90)	
Negative	29 (48)	20 (10)		27 (20)	4 (10)	
PR			<0.001			0.240
Positive	31 (52)	164 (80)		99 (73)	32 (82)	
Negative	29 (48)	41 (20)		37 (27)	7 (18)	
BCL2			0.003			0.494
Positive	40 ()	173 ()		107 (82)	32 (87)	
Negative	17 ()	26 ()		24 (18)	5 (13)	
pT ^a^			0.692			0.858
pTis	0 (0)	1 (1)		1 (1)	0 (0)	
pT1	15 (44)	73 (48)		52 (53)	11 (46)	
pT2	15 (44)	63 (42)		38 (38)	11 (46)	
pT3	4 (12)	10 (7)		6 (6)	2 (8)	
pT4	0 (0)	4 (3)		2 (2)	0 (0)	
pN ^a^			0.478			0.659
pN0	17 (53)	84 (59)		55 (59)	16 (70)	
pN1	10 (31)	49 (34)		32 (35)	5 (22)	
pN2	3 (10)	6 (4)		4 (4)	1 (4)	
pN3	2 (6)	4 (3)		2 (2)	1 (4)	
pM ^a^			0.289			0.476
pM0	31 (100)	137 (97)		90 (98)	23 (100)	
pM1	0 (0)	5 (3)		2 (2)	0 (0)	
cT ^b^			0.716			0.722
cTis	0 (0)	0 (0)		0 (0)	0 (0)	
cT1	2 (9)	2 (7)		3 (10)	0 (0)	
cT2	12 (54)	12 (41)		12 (42)	4 (57)	
cT3	5 (23)	8 (28)		7 (24)	2 (29)	
cT4	3 (14)	7 (24)		7 (24)	1 (14)	
cN ^b^			0.687			0.176
cN0	6 (27)	8 (28)		6 (21)	4 (57)	
cN1	9 (41)	8 (28)		10 (34)	1 (14)	
cN2	3 (14)	4 (13)		6 (21)	0 (0)	
cN3	4 (18)	9 (31)		7 (24)	2 (29)	
cM ^b^			0.823			0.211
cM0	18 (82)	23 (79)		26 (90)	5 (71)	
cM1	4 (18)	6 (21)		3 (10)	2 (29)	
p53			0.962			0.822
Positive	40 (70)	139 (70)		96 (74)	28 (76)	
Negative	17 (30)	60 (30)		34 (26)	9 (24)	
Ki67			0.002			0.048
<14%	11 (18)	82 (40)		47 (35)	7 (18)	
≥14%	49 (82)	123 (60)		89 (65)	32 (82)	
E-Cadherin			0.194			0.989
Positive	52 (90)	166 (83)		118 (89)	34 (90)	
Negative	6 (10)	35 (17)		14 (11)	4 (10)	
Fascin						0.893
High	-	-		30 (22)	9 (23)	
Low	-	-		106 (78)	30 (77)	
TOP2A			0.893			
Altered	9 (23)	30 (22)		-	-	
Normal	30 (77)	106 (78)		-	-	
SBR ^c^			<0.001			0.033
3	0 (0)	9 (5)		5 (4)	0 (0)	
4	4 (7)	24 (12)		14 (11)	3 (8)	
5	3 (5)	40 (20)		24 (18)	6 (15)	
6	12 (21)	65 (32)		32 (24)	15 (38)	
7	17 (29)	36 (18)		24 (18)	13 (33)	
8	18 (31)	24 (12)		29 (22)	1 (3)	
9	4 (7)	3 (1)		3 (2)	1 (3)	
SBR ^c^ per grades			<0.001			<0.001
Grade 1	7 (12)	73 (36)		43 (33)	9 (23)	
Grade 2	29 (50)	101 (50)		56 (43)	28 (72)	
Grade 3	22 (38)	27 (14)		32 (24)	2 (5)	

ER, estrogen receptor; PR, progesterone receptor; TOP2A, DNA Topoisomerase II Alpha. RE, RP, BCL2, and p53 were considered negative when 0, and positive when ≥1. ^a^ Pathological tumor stage, pathological T, pathological N, pathological M, Patients not receiving neoadjuvant therapy. ^b^ Clinical tumor stage, clinical T, clinical N, clinical M, Patients receiving neoadjuvant therapy. ^c^ SBR Scarff–Bloom–Richardson Scale for Histological Tumor Grade.

**Table 3:** Association of studied variables with pathologic complete response in the full series.

Binary Logistic Regression (pCR)	OR	CI 95%	*p*
Univariate analysis			
ER	1.029	1.009–1.050	0.005
PR	1.020	0.996–1.044	0.098
p53	0.989	0.972–1.007	0.239
BCL2	1.024	1.006–1.043	0.010
Ki67	0.957	0.930–0.986	0.003
E-Cadherin	1.917	0.110–33.412	0.656
Histological grade SBR	0.434	0.196–0.963	0.040
Menopause	1.731	0.411–7.288	0.455
Age	1.022	0.941–1.067	0.956
Clinical stage	3.680	1.026–13.197	0.046
Histological subtype	0.871	0.528–1.436	0.588
Molecular subtype	0.169	0.045–0.641	0.009
TNBC/NO TNBC	7	1.350–36.306	0.021
Fascin (+/−)	2.667	0.664–10.704	0.167
TOP2A	0	0–0	0.999
Multivariate analysis			
Clinical stage	9.066	0.875–93.880	0.065
Histological grade SBR	2.576	0.477–13.925	0.272
Molecular subtype	0.037	0.002–0.819	0.037

**Table 4:** Overall survival analysis.

	Chi-Sqr	Degrees of Freedom	*p*
Log Rank (Mantel-Cox)			
**Histological subtype**	21.642	8	**0.006**
**Molecular subtype**	5.857	4	0.210
**CK19 (+/−)**	0.368	1	0.544
**Menopause (Yes/No)**	3.727	1	0.054
**Pathological tumor stage**	14.805	3	**0.002**
**Tumor size (pathological T)**	21.895	4	**<0.001**
**Affected lymph nodes (pathological *n*)**	15.377	3	**0.002**
**Metastasis (pathological M)**	6.477	1	**0.011**
**IHC HER2**	5.382	3	0.146
**E-Cadherin (+/−)**	1.009	1	0.315
**ER (+/−)**	5.456	1	**0.020**
**PR (+/−)**	1.646	1	0.200
**p53 (+/−)**	3.585	1	0.058
**BCL2 (+/−)**	4.192	1	**0.041**
**Ki67(<14%/≥14%)**	3.836	1	0.050
**Age (<65/≥65)**	12.657	1	**<0.001**
**Histological grade SBR (grouped)**	2.844	2	0.241
**TOP2A**	0.034	1	0.853
**Fascin**	1.783	1	0.181
**Cox regression (multivariate analysis)**			
	**HR**	**CI 95%**	***p***
**Histological subtype**	2.437	0.648–9.162	0.187
**Pathological tumor stage**	2.803	1.467–5.356	**0.002**
**ER (+/−)**	3.120	0.157–61.854	0.455
**BCL2 (+/−)**	0.349	0.056–2.190	0.261
**Age (<65/≥65)**	5.098	0.627–41.410	0.128

## Data Availability

The data that support the findings of this study are not openly available due to reasons of sensitivity and are available from the corresponding author upon reasonable request. Data are located in controlled access data storage at Hospital General Universitario Santa Lucía.

## Abbreviations

The following abbreviations are used in this manuscript:

DNA	Deoxyribonucleic acid
TOP2A	Topoisomerase II alpha
ATP	Adenosine triphosphate
CEP17	Centromere Enumeration Probe 17
IHC	Immunohistochemical
HR	Hormone receptor
ER	Estrogen receptor
OS	Overall survival
DFS	Disease-free survival
CNB	Core needle biopsy
VAB	Vacuum-assisted biopsy
NOS	No other subtype
WHO	World Health Organization
CNS	Central nervous system
TDCC	TOP2A-DNA covalent complexes
FISH	Fluorescence in situ hybridization
TNBC	Triple-negative breast cancer
SBR	Scarff–Bloom–Richardson
PR	Progesterone receptor
pCR	Pathological complete response
mRNA	messenger ribonucleic acid
RT-PCR	Reverse transcription polymerase chain reaction
CISH	Chromogenic in situ hybridization
H&E	Hematoxylin and Eosin
AJCC	American Joint Committee on Cancer
